# New Jersey State Dental Society

**Published:** 1890-01

**Authors:** 


### NEW JERSEY STATE DENTAL SOCIETY.

### DISCUSSION ON PROF. MAYR'S PAPER ENTITLED "THE WASTE PRODUCTS OF THE BODY."

*Dr. Sudduth.*—Mr. President, the paper that Professor Mayr has given us has been intensely interesting to me. But to some of you, perhaps, what has been said is more or less Greek. You may have listened to his figures and propositions without a clear understanding of what they indicate, or the exact meaning of the formulæ. It is impossible for a man to grasp in its entirety the subject as given here. Too much stress cannot be laid, however, upon the value of such work as this. Very often, in my practice, I come to a practical application of my scientific studies in the examination of the waste products of the body. We cannot too highly appreciate the rapid advance that has been made in the past few years in methods of making more accurate diagnoses. There is no longer any need of making guesses as to whether a man has a good or a weak digestion, or whether his nutrition is good or deficient, we can locate the very point of the body where the lack of assimilation occurs, make an accurate diagnosis, and administer medicines which will act directly. A chemist of Professor Mayr's standing, or any other man, whose life work has been in this direction, can take the waste products of the body, provided he knows the diet of the patient, his age, the time of day, and the quantity of urine passed daily, and give you a very correct diagnosis of the disease from which the patient is suffering. It has been done time and again, and the result is as accurate as mathematics. The day of accurate diagnoses is here. You should consult specialists in this work. Dentists do not understand the influence and bearing that this character of work will have in giving them accurate bases upon which to argue and to build their theories. I cannot say too much regarding the benefit that will come to the dental profession from this line of investigation. I simply want to make this general application of the technical talk that has been given you. But bear away this thought, that the specialties of the specialists are being brought to the highest state of perfection, and you want to consult them in order to get a solid foundation on which to base your theories.

*Dr. V. H. Jackson.*—There is a practical bearing of this paper upon our work; and as dentists we should search for what can best assist us in our work. All dentists cannot make analyses of urine, or understand urine analyses. Dr. Sudduth has very nicely arranged it for us, in turning the subject over to specialists. I think it is proper that we should consult specialists. I spent three or four months in each of two different courses of study in order to prepare myself to understand this subject. It requires a long course of study; and we should not deceive ourselves into the belief that we can master the subject like our friend, Professor Mayr. My suggestion would be to try to get the most benefit we can out of the subject by dealing with the practical side of it; to try phosphate feeding, and learn in what forms the phosphates are best assimilated. Intricate cases, where there is pyorrhœa, I think we should turn over to the specialist and let him determine it for us. I would like Dr. Mayr's suggestions as to the most practical method of feeding patients that are deficient in phosphates, and how we can promote the hardening of soft tooth-tissue. I have great faith in phosphate feeding.

*Dr. Faught.*—The question resolves itself down to the control of the whole nervous system, as one of the ways to build up the tissues, as much as to administer the proper assimilable material. If we can reduce the waste, we take a great step towards the building up of the tissues. The elimination of urea shows a breaking down of the nervous forces, and the tissues and the phosphates bear their portion of the loss. That being the case, dentists must direct their attention to means of controlling their patients' mental forces. By controlling the nervous forces you will control the loss of urea, and with it the elimination of the phosphates.

*Dr. Littig.*—What portion of the wheat kernel is assimilable in the body,—the phosphite or the phosphate?

*Prof. Mayr.*—I think that where an albuminous substance is digested and taken up by the system, the phosphate in combination with it is also digested and taken up; and phosphoric acid excreted. If we could label the food that goes into the body, we would find that the phosphoric acid that comes out is not the same that went in. It is an old one, perhaps weeks old. The phosphate that is excreted comes from the tissues that have been eaten up, and not necessarily from the food that has recently been digested. The phosphoric acid excreted is not much influenced by a meal that is taken; it depends upon the urea. By examining the urea excreted by a man I can get an exact measurement of his mental energy; I can tell how much a man thinks in the next two hours after eating.

The excretion of urea at different times, according to the activity or inactivity of the mind, is very interesting. At midnight it reaches the lowest point. About three o'clock the subject begins to dream, and the excretion of urea begins to rise. Between three and five o'clock is the time for dreaming; dreams cause thought, and thought is expressed in the urea. Then the person falls into a quiet slumber from five to six o'clock, and the urea falls. From nine to ten o'clock there is hard work, and at twelve o'clock (noon), the urea is tremendously high. It has been formed during the period of work. *The kidneys do not immediately excrete it, but two or three hours after.* At twelve o'clock the man has his dinner. That does not require much thought; and so the quantity of urea found falls off during the dinner hour. Two hours are taken for digestion, and there is not much change. From two to five there is generally hard work; and at six o'clock the urea rises again, and keeps up until seven or eight o'clock. At six o'clock comes supper; after which there is sometimes a good deal of work done, sometimes there is not, but the urea excreted will mark the energy expended.

The phosphoric acid excreted goes exactly parallel with the urea; showing that the phosphoric acid excreted is precisely that acid which goes with the tissue from which the urea is made. I think that is most conclusive proof that it is not the phosphoric acid put into the system in the food. If it were, it would be high after breakfast and high after dinner, which is not the case. The phosphoric acid that we excrete is made from the tissue. The urea is made from the brain, and is excreted in the kidneys. The uric acid is entirely different. It has nothing to do with the urea; but has a great deal to do with the digestive process. The urea is lowest after every meal; the uric acid is highest after dinner and after breakfast; three or four hours after a meal it shows the highest. The salt, on the other hand, which is purely a waste product, shows the influence of the meals in a most striking manner. In it you can see exactly the speed of digestion. The excretion of salt shows that it comes from the meals taken, and does not necessarily come from the tissues of the body.

There are many other constituents of the urine that I have omitted. I have made thousands of careful examinations, and given this subject much study. I think there is conclusive proof that the phosphoric acid we excrete is not the particular one we take into the system in our food. The phosphoric acid which we take in as phosphate has to be worked over, and the phosphate excreted is from the tissue burned up.

*Dr. Sudduth.*—It was stated, in the discussion on the treatment of the teeth during pregnancy, that pregnant women crave lime and chalk; and the question was raised whether it was the phosphate or the phosphite that the system demanded and assimilated.

*Prof. Mayr.*—I should say the phosphate; because the phosphite, as a distinct chemical compound, is not found in the body.

*Dr. Rhein.*—In connection with Dr. Sudduth's question, I would like to ask Professor Mayr's opinion about the action of a preparation that has been very widely recommended,—that is, in regard to the various compounds of hypophosphites mixed with lime and other ingredients. I have heard it claimed that the phosphates and lime salts were assimilated when taken in that way.

*Prof. Mayr.*—I have made experiments on that subject; and there seems to be proof that the phosphates, etc., are excreted just as they are taken in. By analysis you can recover everything again. The hypophosphites appear mostly as phosphates, but not altogether. A certain amount seems to pass through the alimentary canal as hypophosphites; but they do not seem to be assimilated. When five grains of phosphate are taken five grains will invariably be excreted in excess of the ordinary proportion. The administration of hypophosphites seems to have a good influence in certain cases of defective digestion. They act merely as a tonic, such as the subnitrate of bismuth.

*Dr. Meeker.*—Is the preparation called "Horsford's Acid Phosphates" excreted in the same form?

*Prof. Mayr.*—It is excreted, as a rule, in the form of phosphoric acid. It is a mixture of pyro- and ortho-phosphoric acids.

*Dr. Meeker.*—Is not a portion assimilated?

*Prof. Mayr.*—In my opinion it is not; not the least particle of it.

*Dr. Thayer.*—Did you ever test the urine of persons whose bread foods are confined entirely to the products of unbolted wheat flour and compare the results with those obtained in cases where the bread food had been made of fine flour only?

*Prof. Mayr.*—Not sufficiently to form any definite conclusions. I do not always have the right subjects and circumstances for such tests. I have made tests in cases of vegetarians, those who avoid all kinds of meat, and the ratio is the same. It makes no difference whether the person is a beef-eater or a bean-eater; the results are the same.

To make such investigations of real value, the total urine voided in a day should be mixed and the examination made from that. The weight of a person does not bear any relation to the quantity of urea excreted. A short person with an active brain burns up as much or more than a tall and sluggish person. I have made careful examinations in persons where violent exercise had been taken; and, on the other hand, of persons who did not exercise much but thought a great deal; and I found that the man who thought beat the athlete in the production of urea by long odds, although he was of lighter weight. The athlete evidently used up the fatty constituents more; the other one did not assimilate the same amount of fat, and did not care for it, but he burned the saccharine constituents and the nitrogeneous tissues.

*Dr. Stockton.*—Where there is waste of the tooth-structure from some cause, can that be remedied by taking a particular kind of food to supply that waste?

*Prof. Mayr.*—Certainly. My opinion is that it can: but the food has to be prepared in a form in which it is naturally and normally taken. One person won't eat bread; another person won't have meat; or, if they take it, the digestive apparatus don't work it right. Sometimes there is a defect in the stomach, sometimes in the absorbent qualities; very often the food is not presented in palatable form owing to bad cooking; and sometimes they don't like, or will not appropriate, a particular article of food. I think that bread made of the entire wheat kernel is by far the best kind for supplying the phosphates. There is a good amount of indigestible matter in it; but the intestinal tract requires to be filled up; therefore the waste matter is necessary. We do not want to live on the most concentrated food; we are not built on that plan. The twenty-two feet of intestine has to be filled up. Pig's feet, which contain a great deal of gristle, are sometimes an acceptable food and sometimes not; veal, in various forms, is also a very good food. Veal contains a large amount of phosphates and formative tissue. I think children are not usually given enough veal. It is considered a sort of unripe meat; but children like unripe things; they are unripe themselves, and unripe things are sometimes good for them.

*Dr. Littig.*—It was stated that the urea was made by the brain and excreted by the kidneys. Is it not elaborated in the kidneys; not excreted there?

*Prof. Mayr.*—My conclusion would be contrary to that: unless we suppose the kidneys were closely occupied with the thinking process, and that the brain is only an appendage of the kidneys. Urea circulates in the blood; and you purchase it in the stores as extract of beef, which is concentrated urine. It contains some salts that are very valuable, as creatine and creatinine; both are found in the muscular tissue which we eat. It is excreted, apparently, in the same quantity as taken in. It has a very pleasant taste, and stimulates digestion. The process of carrying the urine and its salts and filtering it through the epithelia of the kidneys is a very complicated one. I do not think the kidneys manufacture anything, in any important sense. They may contribute towards the formation of uric acid, or some of the less known constituents of the urine.

*Dr. Rhein.*—Judging from the scale you have on the black-board, is it not a good, practical conclusion to come to, that where examinations are made for the purpose of determining the existence of pathological conditions, it is always necessary to make several examinations of the urine excreted at different times?

*Prof. Mayr.*—Certainly. This scale was obtained by the examination of probably thirty specimens a day for several weeks.

*Dr. Rhein.*—You would not make a diagnosis in any individual case from a single specimen?

*Prof. Mayr.*—Certain pathological products occur at all times, and a general diagnosis may be obtained from random samples, but nice work could not be so done. In the case of a man who sleeps during the day and works during the night, the result would be totally reversed. I could tell his work if I had a sample of his urine and the time when taken.

*Dr. Rhein.*—I mean in pathological conditions; would it not be preferable not to depend on a single secretion, at a single hour?

*Prof. Mayr.*—That is a point on which I have had to fight a good deal. Very often physicians send me specimens of urine without saying when it was voided, or how old the specimens were or anything. Without that information it is very difficult to form an intelligent opinion. In the morning the urine shows best the excretion of urea and salts and such things; but it is not conclusive in cases of diabetes and Bright's disease. In the evening, and up to midnight the urine will best show those disturbances. In these hours the disease is aggravated. Of course, an examination of the urine voided every hour would lead to more reliable conclusions.

*Dr. Rhein.*—My plan is to take the morning urine, the mid-day, and just before retiring, and make a comparison of the three specimens. I would like to know whether that is sufficient to form correct conclusions regarding pathological conditions?

*Prof. Mayr.*—I think that would give a very fair idea.

*Dr. Faught.*—Mr. President, I would like to state a case in which I am much interested, and which has direct reference to practitioners of dentistry. A dentist who worked very hard at his profession during the day, and mentally at night, for a number of years, passed phosphates in large quantities through the urine. He did not seem to suffer physically in any way from it, but increased in weight. At that time an examination of his mouth revealed no salivary deposits. After quite a long period the secretion or elimination of phosphate through the urine ceased; and then he immediately suffered from severe inflammation of the kidneys, particularly on the left side. I thought that the condition might be due to the vascular supply on the left side being different from that on the right side. He also suffered immediately with hemorrhoids and varicocele. Probably these conditions were aggravated by his work at the chair. Albumen was found in the urine in appreciable quantities. This condition continued for a period of three to five weeks. At the end of that time the albumen disappeared from the urine, under treatment; and immediately upon his being pronounced well he began to throw off phosphates in large quantities again. During the five or six weeks when he was suffering from these troubles there was in his mouth a very great deposit of calculus.

*Prof. Mayr.*—That is a very interesting case. He "gained weight." But what is weight in our bodies? The greater part of it is nothing but water. You often find that we increase in weight while we lose strength. We pump water into our systems, but it does not give us strength. Gain in weight is not necessarily a gain in strength. In regard to the excretion of phosphates in the case mentioned, I would ask what the figures were as to the phosphates obtained?

*Dr. Faught.*—I have no statistics in the case.

*Prof. Mayr.*—We have first to inquire whether it was really so. The description of the case would seem to be indicative of a clogging up, or diphtheria, of the tubes of the kidneys. It is quite correct to say diphtheria of the kidneys. I have a case where the kidneys refused to work; and upon treatment with turpentine there came violent spasms; and I found in the first urine voided a perfect tree of algæ-like threads (plants), showing the ramifications of the tubuli of the kidneys. It was a fungus which had grown up in the tubuli of the kidney. The action of the turpentine enabled the kidney to throw it off, and the patient was cured. I think the case which the gentleman mentioned here might have been a case of that kind.

*Dr. Peirce.*—Yesterday we had very beautifully shown the effect of over-work upon the teeth of young misses in the public schools, and Professor Mayr has brought out a similar thought in regard to mental work generally. I would like to have him speak more at length in connection with those points.

*Prof. Mayr.*—I do not think you will fail to agree with me. The more mental work, the more urea is produced. Young misses occasionally over-work, but a boy seldom over-works himself. You have this state of affairs: people while thinking are continually burning up their tissues for the purpose of producing urea and phosphoric acid. They excrete the phosphoric acid set free from their tissues. They produce too much urea, and are therefore compelled to excrete too much phosphoric acid; because the proportion of one hundred to eight is maintained; therefore, as they cannot think, and especially worry, without producing much urea, they have to excrete a corresponding amount—the constant ratio of one hundred to eight—of phosphoric acid. For every foot-pound of thought you will have a given amount of urea excreted; therefore, as long as you keep the brain at work and make it perform so many foot-pounds of thought, you will have excreted so many grains of urea. One thing is to control fretfulness. A fretfulness that produces activity, but no actual results, causes a loss of just so many grains of urea. Another wearing cause may be intestinal parasites; or the limbs may be moving around uneasily, caused by a tendency to epilepsy or St. Vitus's dance. A peculiar fretfulness of the nerves produces work that is not work, but which turns out urea just the same.

*Dr. Jackson.*—Are we to understand the doctor to imply that phosphate feeding is not of advantage to the patient?

*Prof. Mayr.*—My opinion would be strongly against feeding phosphates pure,—that is, not in combination with the albuminoids, such as are accepted by the body. The phosphate in wheat is actually in such chemical combination. We have not been able to produce that combination chemically. We have been able to dissolve it. Take the phosphates away from the albuminous substance and it becomes an entirely different compound. It is no longer soluble in water. Dogs have been starved by feeding them on the cakes from which the extract of meat is obtained. Those cakes were like so much rubber, and the dogs starved on it.

